# Building a Lung Transplant Program

**DOI:** 10.6061/clinics/2015(12)02

**Published:** 2015-12

**Authors:** Marcos Naoyuki Samano, Paulo Manuel Pêgo-Fernandes

**Affiliations:** Hospital das Clínicas da Faculdade de Medicina da Universidade de São Paulo, Instituto do Coração (INCOR), Divisão de Cirurgia Torácica, São Paulo/SP, Brazil.

Twenty-five years have passed since the first lung transplant was performed at the Instituto do Coração (INCOR) do Hospital das Clínicas da Faculdade de Medicina da Universidade de São Paulo. On October 16^th^, 1990, a 26-year-old woman with severe pulmonary fibrosis and secondary pulmonary hypertension underwent a single left lung transplant but only survived for 12 days, dying due to sepsis and diffuse alveolar damage. This occurred several months after the first and second 1 lung transplants in Brazil and seven years after the first in the world, which had been performed by Dr. Cooper in Toronto 2. Over a five-year period, nine more cases were performed, with the longest post-operative survival being 4 years. Despite the dedication of all the personnel involved with the program at that time, seven patients were never discharged due to acute respiratory failure and infection. At present, primary graft dysfunction remains a complication of transplant procedures, but in 1995, it seemed an almost insurmountable barrier. Despite the strong desire to continue performing this procedure, the group was not adequately prepared to continue in this field and the program was discontinued.

In parallel, pulmonologists and surgeons sought training at other lung transplant centers and research in the field was initiated at our laboratory in the Medical School. Because bronchial healing remained a problem in clinical cases, it was chosen as a starting point for research. Using animal models of bronchial anastomosis and lung transplant as well as an established methodology for the evaluation of airway mucous clearance, the effect of immunosuppressive drugs was tested. Cyclosporin 3,5, Prednisone 6,7, Micophenolate 8 and combined therapies, including Tacrolimus 9,10, were tested and all drugs had a negative impact on mucous clearance.

On August 9^th^ 2000, a single right lung transplant was performed on a 32-year-old male with silicosis. The post-operative period was uneventful and the patient is still alive fifteen years later. The main problems concerning intraoperative care, post-operative care and immunosuppression seemed to be solved. However, in the following three years we performed ten more procedures, of which only two patients survived for more than one year. Although only two patients of this period are still alive, the low survival rate indicated that there was still something lacking. At that time, the current knowledge and infrastructure were considered sufficient, but it was clear that unless a dedicated team was established, we could not proceed in this field.

In 2003, a multi-professional and multi-disciplinary team was formed that included pulmonologists, surgeons, infectologists, nurses, physiotherapists, nutritionists, social workers and psychologists. We established a routine of morning rounds in ICU and on the wards; assessment meetings; scientific meetings and journal clubs. Based on candidate selection criteria established by the International Society for Heart and Lung Transplantation, patients considered for LTx were assessed and visited by all members of the lung transplant team. Inclusion on a waiting list was possible only upon the agreement of all members of the staff.

Since then, almost 250 procedures have been performed, including some landmark procedures: the first bilateral procedure in 2003, the first pediatric transplant in 2006, the first split transplant in 2011 and the first transplant using the ex vivo lung perfusion (EVLP) technique in 2012. However, some problems persisted. Initially, simple preoperative monitoring and patient positioning were problematic and each transplant presented individualized challenges, illustrating the difficulties inherent in establishing routines in the operating room.

However, over time, the program has achieved consistently satisfactory results and survival in line with international rates (Figure 1). For diseases such as cystic fibrosis, the survival to the end of the first year is over 90%, even higher than the ISHLT Resistries 11. Approximately 35 procedures are performed annually in the State of São Paulo - a modest number compared to the number of patients on the waiting list, which impacts on the mortality rate of patients on the list. The main reason for the low number of transplants performed is the low success rate of lungs obtained from multiorgan donors. It is estimated that only 5% of available lungs are used, a very low frequency compared to the average rate of 15% reported by the UNOS 12. The main reason for the low utilization is the quality of the available lungs, which are rejected more than 50% of the time due to infection or inadequate management resulting in loss of lung function 13.

Improving the quality of available donor lungs seemed to be the best solution for increasing the number of transplants. Thus, the first report on the reconditioning of initially rejected donor lungs by *ex vivo* perfusion 14 presented a solution for this problem. In 2007 we began preparations for EVLP via training courses and experimental studies with lungs rejected for transplantation. We initially sought to establish our methodology 15,16, then tested lung preservation solutions 17 and static or continuous topical preservation methodologies 18. This prepared us to perform the first lung transplantation using EVLP in Brazil in 2012. Three years after the procedure, the first patient is doing well. However, the cost and quality of using EVLP donor tissue in our country, and as a result, other transplants have not been performed with this technique. New research has been implemented in this area that incorporated the use of broad spectrum antibiotics during EVLP 19 and in trachea transplantation 20,21.

Two important issues remaining are the training of personnel and international recognition. All staff members were trained in our institution and completed the training in transplant centers in North America or Europe. The Ministry of Health has certified InCor for medical residency in pulmonary and surgical transplantation, as well as two years of training in a clinical or surgical fellowship in lung transplantation certified by the Ministry of Education. We are internationally recognized as a part of the ISHLT registry and we actively participate in international congresses as members of the society and through the submission of scientific papers.

Institutional support is fundamental for the maintenance of staff and we were supported without fail throughout the formation of the transplant group. Although the number of transplants performed every month remains small, the need of these patients for care is intense. Thus, maintaining a professional team on a full-time basis will only be feasible with professional development and salary adjustments. In 2013, the Transplant Center Unit of InCor was created, which relies on a 24/7 nursing team fully dedicated to procuring thoracic organs and mediating and enhancing the work of the transplant teams, resulting in the realization of 98 transplants in 2014, including adult and pediatric heart transplants and lung transplants.

The road traveled thus far has enabled the formation of an accomplished group and national leadership in this area. It is essential that the foundations of our group are the same as those of universities: care, teaching and research. We believe that we have achieved these goals, but much remains to be accomplished in this field.

## Figures and Tables

**Figure 1:**
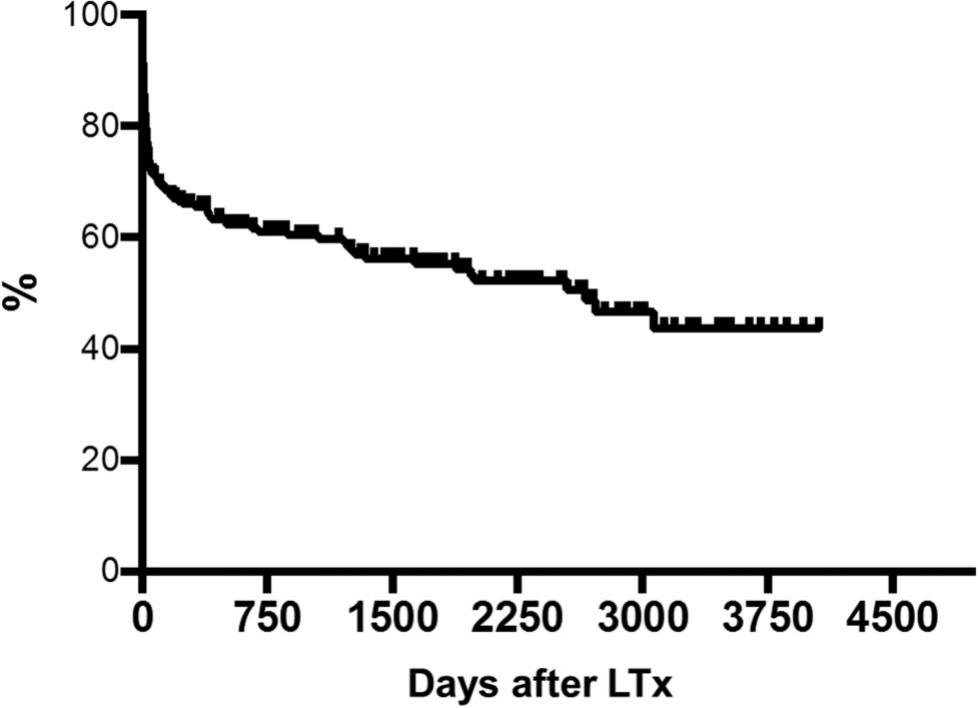
Kaplan-Meier Survival Curve of LTx patients. One year survival: 65.6%, Five years survival: 55.3% and Ten years survival: 43.7%.
